# Somatostatin Therapy Improves Stellate Cell Activation and Early Fibrogenesis in a Preclinical Model of Extended Major Hepatectomy

**DOI:** 10.3390/cancers13163989

**Published:** 2021-08-07

**Authors:** Amelia J. Hessheimer, Jordi Vengohechea, Lilia Martínez de la Maza, Javier Muñoz, Marina Vendrell, Josep Martí Sanahuja, Alba Torroella, Farah Adel Al Shwely, Francisco Riquelme, César Muñoz, Rocío García, Pilar Taurá, Constantino Fondevila

**Affiliations:** 1 General & Digestive Surgery Service, Hospital Clínic, 08036 Barcelona, Spain; AJHESSHE@clinic.cat (A.J.H.); lilia.martinez-delamaza@sll.se (L.M.d.l.M.); torroella@clinic.cat (A.T.); farahadeladel@gmail.com (F.A.A.S.); frriquelme@uchile.cl (F.R.); cesarmunozcastro@gmail.com (C.M.); RGARCIA5@clinic.cat (R.G.); 2 CIBERehd, IDIBAPS, University of Barcelona, 08036 Barcelona, Spain; jvengo@clinic.cat (J.V.); javier.munoz@ciberehd.org (J.M.); 3 Anesthesiology, Hospital Clínic, University of Barcelona, 08036 Barcelona, Spain; mvendre1@clinic.cat (M.V.); sanahuja@clinic.cat (J.M.S.); ptaura7@gmail.com (P.T.)

**Keywords:** pig, liver resection, small-for-size syndrome, post-hepatectomy liver failure

## Abstract

**Simple Summary:**

Liver resection is the best treatment for liver tumors; its clinical applicability, however, is limited by the mass and quality of the remnant liver. In this study, hepatic resections were performed in a pig model to define changes associated with sufficient and insufficient liver remnants. As well, somatostatin, a naturally occurring peptide hormone that exerts numerous largely inhibitory effects, was given in order to improve outcomes. We observed that extended major hepatectomy is associated with immediate changes in portal pressure leading to injury, stellate cell activation, collagen deposition, and cell death, all of which were improved with somatostatin therapy. This is one of few studies (potentially the first) describing collagen deposition as early as 24 h after extended major hepatectomy and implicating liver resection as potential cause for chronic liver injury.

**Abstract:**

Liver resection treats primary and secondary liver tumors, though clinical applicability is limited by the remnant liver mass and quality. Herein, major hepatic resections were performed in pigs to define changes associated with sufficient and insufficient remnants and improve liver-specific outcomes with somatostatin therapy. Three experimental groups were performed: 75% hepatectomy (75H), 90% hepatectomy (90H), and 90% hepatectomy + somatostatin (90H + SST). Animals were followed for 24 h (N = 6) and 5 d (N = 6). After hepatectomy, portal pressure gradient was higher in 90H versus 75H and 90H + SST (8 (3–13) mmHg vs. 4 (2–6) mmHg and 4 (2–6) mmHg, respectively, *p* < 0.001). After 24 h, changes were observed in 90H associated with stellate cell activation and collapse of sinusoidal lumen. Collagen chain type 1 alpha 1 mRNA expression was higher, extracellular matrix width less, and percentage of collagen-staining areas greater at 24 h in 90H versus 75H and 90H + SST. After 5 d, remnant liver mass was higher in 75H and 90H + SST versus 90H, and Ki-67 immunostaining was higher in 90H + SST versus 75H and 90H. As well, more TUNEL-staining cells were observed in 90H versus 75H and 90H + SST at 5 d. Perioperative somatostatin modified portal pressure, injury, apoptosis, and stellate cell activation, stemming changes related to hepatic fibrogenesis seen in liver remnants not receiving treatment.

## 1. Introduction

Globally, liver cancer is a leading cause of cancer-related death. According to Global Cancer Statistics 2020, primary liver tumors were the seventh most frequent cancer diagnosis (905,677 new cases) and second after lung cancer as a leading cause of cancer death [1]. As well, colorectal cancer was the fifth most frequent cancer diagnosis (1,148,515 new cases), and it is known that 30–50% of these patients will develop liver metastases over the course of their disease process [1,2].

First-line therapy of both primary and secondary liver tumors involves surgical resection of the affected parenchyma, including the tumor in its entirety along with a margin of non-tumoral tissue. Surgical and technological advances have allowed for hepatic resection to become a relatively routine procedure in modern surgical practice. Knowledge of hepatic anatomy combined with the use of intraoperative ultrasound allow for parenchymal transection to proceed while minimizing blood loss and preserving vascular and biliary structures to the remnant liver [3]. The limiting factor in performing hepatectomy remains leaving enough mass of sufficient quality to avoid development of liver dysfunction and failure in the postoperative period.

Post-hepatectomy liver failure (PHLF), also known as “small-for-size” syndrome (SFSS, the latter more typically in the context of partial liver transplantation), is characterized by progressive cholestasis, coagulopathy, encephalopathy, ascites, and even renal and/or respiratory failure. While milder presentations include laboratory abnormalities requiring no specific treatment, severe cases require intensive care management, various forms of life support (hemodialysis, mechanical ventilation, and occasionally extracorporeal liver support or rescue liver transplantation) and are associated with short-term mortality rates of up to 80%. Careful surgical planning is critical to ensure safe graft or future liver remnant >25–30% of its preoperative mass or volume in patients with normal livers or >40% in livers that are cirrhotic, cholestatic, steatotic, or injured by chemotherapy [4,5,6,7].

Upon reduction of liver mass, portal vein flow (PVF) to the remnant liver increases, exerting mechanical effect in the sinusoids and bringing blood with higher a volume of signaling molecules to the hepatocytes. Subsequent intra- as well as extracellular signaling leads to changes in gene and protein expression and re-entry into the cell cycle by previously quiescent hepatocytes and non-parenchymal cells. Normal liver tissue is capable of supporting up to 2–3 times its standard PVF, and this increase is an important stimulus for hepatic regeneration [8,9,10]. Portal vein flow in excess of this threshold, however, can be detrimental to liver function and survival and significantly increases risk for the development of PHLF/SFSS in the postoperative period [10,11,12,13,14,15]. This is due not only to mechanical shear-stress-induced injury to cells lining the hepatic sinusoids, including liver sinusoidal endothelial cells (LSECs) and hepatic stellate cells (HSCs) [14], but also disproportionate restoration of parenchymal versus non-parenchymal cells during the process of regeneration [16].

Somatostatin (SST) is a naturally occurring peptide hormone that exerts a number of effects in living organisms, the majority of which are inhibitory. Due to its specific actions in the splanchnic vasculature, it has found a role in ameliorating adverse effects of hyperdynamic splanchnic and portal vein flow in different clinical situations [17]. For this reason, somatostatin and its analogues have gained increasing interest as a means to modify PVF in liver resection and transplantation, including in the treatment of PHLF (NCT04010669, NCT01290172, NCT02882347, NCT04107428, and NCT02799212).

In the present study, we performed major and extended major hepatic resections in pigs, in order to clearly define the hemodynamic, histological, functional, and genetic changes associated with hepatic remnants of both sufficient and insufficient masses. Moreover, by continuously administering somatostatin throughout the immediate postoperative period, we aimed to improve liver morphology and function as well as liver-specific outcomes.

## 2. Materials and Methods

### 2.1. Animals

Male weanling Landrace-Large white hybrid pigs were used (30 kg, N = 36). Animals were housed and procedures conducted in the Animal Experimentation Unit at the University of Barcelona Medical School and in accordance with current national and European regulations. The Animal Experimentation Unit is authorized by the Catalan Department of Agriculture, Husbandry, and Fisheries (authorization number B9900020), registered in the General Registry of Livestock Facilities (ES080190036536), and accredited by the International Standards Organization (ISO 9001:2015). Animals were cared for according to the guidelines of the Catalan Department of the Environment Commission on Animal Experimentation and University of Barcelona Committee on Ethics in Animal Experimentation, which approved the study protocol prior to its initiation.

### 2.2. Study Overview

Three experimental groups were performed. Animals were randomized to each group using sealed envelopes opened at the start of each experiment. Taking into consideration porcine hepatic anatomy and typical relative mass of each anatomical segment and the fact that weanling pigs sustain greater resections and smaller liver remnants relative to humans [18,19,20], left trisectionectomy (removing segments II, III, IV, V, and VIII and leaving segments I, VI, and VII) was used as the 75% major hepatectomy model and left trisectionectomy plus right lateral sectionectomy (leaving only segment I and a small cuff of segments VI-VII) as the 90% extended major hepatectomy model.

- 75% hepatectomy (75H): left trisectionectomy and follow-up for 24 h (N = 6) and 5 days (N = 6);
- 90% hepatectomy (90H): left trisectionectomy + right lateral sectionectomy and follow-up for 24 h (N = 6) and 5 days (N = 6);
- 90% hepatectomy + SST (90H + SST): left trisectionectomy + right lateral sectionectomy + continuous perioperative infusion of SST and follow-up for 24 h (N = 6) and 5 days (N = 6).

### 2.3. Preoperative Care, Anesthesia, and Monitoring

Animals were acclimated preoperatively, with free access to dry food and water. They were maintained in pens in a room with an ambient temperature of 15–18 °C and alternating 12 h light–dark cycles. Twelve hours prior to induction, food was withheld. Anesthesia was induced preoperatively with a mix of ketamine (3 mg/kg, i.m.), xylazine (2.5 mg/kg, i.m.), and midazolam (0.17 mg/kg, i.m.). Anesthesia was induced through a peripheral ear vein with sodium thiopental (5 mg/kg, i.v.), followed by endotracheal intubation. The pig was ventilated with volume-controlled intermittent positive pressure ventilation, and anesthesia was maintained with isoflurane (1–2%, titrated to effect). Prior to skin incision, fentanyl (100 μg followed by 50 μg/h, i.v.) was administered for analgesia, cefoxitin (1 g, i.v.) for antibiotic prophylaxis, and cisatracurium (0.2–0.3 mg/kg followed by 1.5 mg/h, i.v.) for paralysis. Normovolemia was maintained with the administration of warm crystalloid and colloid solutions.

After open cutdown of the right carotid sheath, a triple lumen catheter was placed in the internal jugular vein for invasive venous pressure monitoring. The catheter was tunneled subcutaneously to exit at the back of the neck for postoperative access.

### 2.4. Surgical Procedure

After opening the abdomen with a “J” shaped incision, the liver and hilar structures were dissected, and cholecystectomy was performed. A single lumen catheter was introduced into the portal vein to monitor portal venous pressure. Ultrasonic flow probes were connected to a flowmeter (HT107, Transonic Systems, Ithaca, NY, USA) to measure hepatic artery flow (HAF) and PVF. The portal venous pressure, HAF, and PVF were recorded in the donor at the baseline and in the recipient after both portal and arterial reperfusion. At these same times, the internal jugular venous catheter was momentarily advanced into the suprahepatic inferior vena cava to record the suprahepatic venous pressure. The portal venous pressure gradient (PVPG) was calculated as the difference between the portal and suprahepatic venous pressures.

Following completion of hemodynamic measurements, the left portal vein was dissected and referenced, at which point a bolus of synthetic 14-amino acid somatostatin (GP Pharma, Barcelona, Spain) was given intravenously (15 μg/kg) in the 90H + SST group or a similar volume of normal saline in the 75H and 90H groups. The left portal vein was then ligated, and hepatectomy was performed using crush-clamp technique, without the Pringle maneuver.

At the end of the surgical procedure, a Surefuser Plus intravenous infusion pump (Nipro Europe, Madrid, Spain) was connected to a lumen of the central venous catheter. The pump consisted in a reservoir, which contained a solution mixed to a predetermined concentration in order to continuously provide 8 μg/kg/h of somatostatin for 24 h or 5 days (90H + SST) or a similar volume of normal saline (75H and 90H).

### 2.5. Postoperative Follow-Up

Postoperative care has been described previously [10]. At the end of follow-up (24 h or 5 days), the pig was anesthetized and reopened and hemodynamic parameters reevaluated. The remnant liver was removed, weighed, and sampled, and the pig was euthanized.

### 2.6. Serum Biochemical Analyses

In blood samples collected serially during follow-up, aspartate aminotransferase (AST), total bilirubin, and the Quick prothrombin time (QPT) were determined using the Advia 1650 automatic analyzer (Bayer, Tarrytown, NY, USA).

### 2.7. Tissue Analyses

Liver tissue samples were collected at baseline (prior to resection) and at 24 h and 5 days. Samples were divided into three sections—one preserved in 10% formalin for paraffin inclusion, one embedded in Tissue-Tek OCT compound (Sakura Finetek Europe B.V., Zoeterwoude, The Netherlands) and snap-frozen in liquid nitrogen, and one diced finely and snap-frozen in liquid nitrogen for mRNA extraction.

Formalin-fixed samples were processed for routine histology. Sections were stained with hematoxylin & eosin (H&E) and Masson’s trichrome (MT) and examined using standard light microscopy. In each sample, sinusoidal congestion and hepatocellular vacuolization and necrosis were assessed according to the Suzuki score [21].

### 2.8. Hepatocellular Proliferation and Apoptosis

Proliferative hepatocyte nuclei were quantified via staining with anti-Ki67 (ab15580, Abcam, Cambridge, UK), while apoptosis was analyzed via protein expression of Bax (bs-0127R, Bioss Antibodies, Woburn, MA, USA) and Bcl-2 (bs-4563R) as well as TUNEL (Sigma-Aldrich Corporation, St. Louis, MO, USA). For TUNEL, paraffin-embedded sections were processed; DNase I-incubated and Tdt-free samples were used as positive and negative controls, respectively. After incubation for 1 h in the dark, samples were co-stained with DAPI and prepared for fluorescence microscopy using Fluormont-G^®^ mounting media (South Biotechnology Associates, Inc., Birmingham, UK). DAPI and TUNEL images at 400× magnification were merged in order to evaluate apoptotic cells.

### 2.9. Hepatic Stellate Cell Activation, Collagen Deposition, and Changes in the Extracellular Matrix

In the quiescent phenotype, hepatic stellate cells (HSCs) are typically localized in the perisinusoidal spaces of Disse, between endothelial cells and hepatocytes. In response to injury, these cells are activated and produce alpha-smooth muscle actin (α-SMA) and collagen, among other molecules.

Levels of α-SMA protein expression in hepatic tissue were determined via Western blot. Whole protein was extracted from tissue samples with a lysis buffer. After protein quantification with DC Protein Assay Reagent B (Bio-Rad Laboratories, Inc., Irvine, CA, USA), samples were prepared with sample buffer and charged in a 12% acrylamide gel. Proteins were transferred to a polyvinylidene fluoride membrane, blocked for 1 h with TBS-T 5% BSA, and incubated overnight with 1/200 α-SMA primary antibody (M0851, Dako-Aligent Technologies, Santa Clara, CA, USA). Membranes were subsequently cleaned and incubated for 1 h with secondary HRP-labeled antibody and visualized using Lumi-Light Western Blotting Substrate (Hoffman-La Roche, Basel, Switzerland). For quantification using ImageJ software, β-actin (4967s, Cell Signaling Technology, Inc., Danvers, MA, USA) was used as housekeeping gene. Results were expressed as relative optical density.

For immunohistochemistry, 3 μm paraffin-embedded sections were cut, dewaxed with xylol, rehydrated with ethanol, and subjected to heat-induced antigen retrieval. Sections were cleaned and blocked for 10 min with EnVision^TM^ FLEX Antibody Diluent (Dako-Aligent Technologies), followed by overnight incubation with 1/200 α-SMA primary antibody. Samples were then incubated 1.5 h with secondary HRP-labeled antibody and visualized with DAB+ Substrate Chromogen System (Dako-Aligent Technologies).

For collagen staining, Picro Sirius Red (PSR; Sigma-Aldrich Corporation, St. Louis, MO, USA) was used in paraffin-embedded sections following dewaxing and hydration for 2 h. Samples were then dehydrated and prepared for light microscopy using DPX mounting media. Positive-staining areas and collagen width were used to assess changes in collagen production and deposition and extracellular matrix (ECM) remodeling. Semi-quantitative analysis was performed by determining the percentage of α-SMA-positive HSCs in nine different high-power fields (HPFs) using 400× magnification.

Total mRNA was extracted in TRIzol^TM^ (Invitrogen, Carlsbad, CA, USA), and real-time quantitative TaqMan reverse transcriptase polymerase chain reaction (RT-PCR) analyses were performed for genes encoding for α-SMA (alpha actin 2, ACTA2), transforming growth factor beta 1 (TGF-β1), and collagen chain type 1 alpha 1 (COL1A1) (ThermoFisher Scientific, Waltham, MA, USA). Gene expression was normalized in each sample relative housekeeping gene HPRT and expressed as fold-change.

### 2.10. Liver Sinusoidal Endothelial Cell Injury

Cluster of differentiation molecule 31 (CD31) immunoglobin helps maintain endothelial stability by interdigitating with other CD31 molecules at the extracellular borders of adjacent cells. Cryosections of hepatic tissue were immunostained with porcine anti-CD31 antibody (MCA1746, Bio-Rad Laboratories) to evaluate the integrity and contraction of LSECs. Samples were photographed and analyzed using ImageJ software.

### 2.11. Data and Statistical Analyses

Values are expressed as mean ± standard deviation or median (25–75% interquartile range), unless otherwise specified. Differences among groups in continuous variables were compared using one-way ANOVA. The Kruskal–Wallis non-parametric test was used for variables with non-Gaussian distributions and the Welch and Brown–Forsythe ANOVA tests for variables with unequal variances. Tukey’s, Dunn’s, and Dunnett’s multiple comparisons tests were used to compare different data sets within each group following ANOVA, Kruskal–Wallis, and Brown–Forsythe tests, respectively. A two-tailed *p* < 0.05 was considered significant. Calculations were performed using GraphPad Prism Software (GraphPad Software, LLC, San Diego, CA, USA).

## 3. Results

All animals survived the surgical procedures and corresponding follow-up period (24 h or 5 days).

### 3.1. Hepatic Hemodynamic Parameters

There were no differences among the groups in any hemodynamic parameters measured at baseline. At the end of surgery, PVPG was higher in 90H but similar in 75H and 90H + SST. After 5 days of follow-up, PVPG remained higher and HAF was lower in the 90H group, though differences with the other two groups were not significant (Table 1).

### 3.2. Hepatocellular Injury

**Figure 1:**
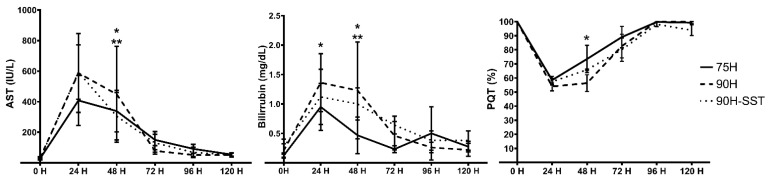
Postoperative evolutions of serum aspartate aminotransferase (AST) and bilirubin and the Quick prothrombin time (QPT) following 75% hepatectomy (75H), 90% hepatectomy (90H), and 90% hepatectomy with continuous perioperative somatostatin infusion (90H + SST). *p* < 0.05 for 75H vs. 90H (*) and 75H vs. 90H + SST (**).

Postoperatively, serum AST and total bilirubin levels increased progressively for the first 24 h, while QPT simultaneously decreased. All parameters subsequently improved back to baseline by 5 days. The bilirubin peak was significantly higher in 90H relative to the other two groups (*p* = 0.03) (Figure 1).

**Figure 2:**
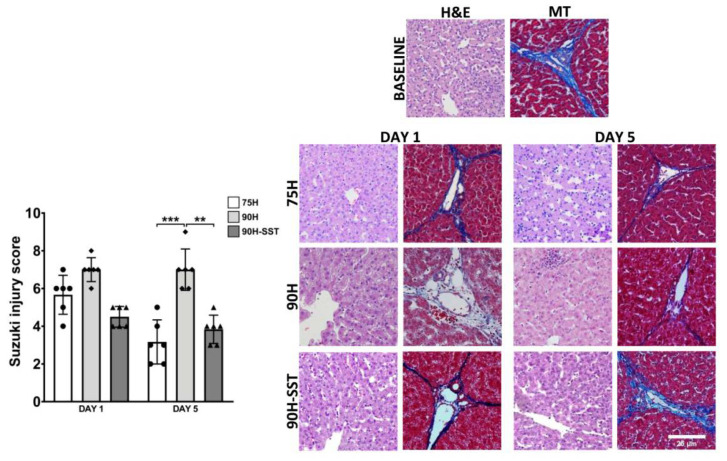
Liver tissue samples stained with H&E and Masson’s trichrome (MT) taken at baseline and 1 and 5 days after 75% hepatectomy (75H), 90% hepatectomy (90H), and 90% hepatectomy with continuous perioperative somatostatin infusion (90H + SST). Changes related to sinusoidal congestion and hepatocellular vacuolization and necrosis were analyzed according to the Suzuki score. Magnification 200×. ** *p* < 0.01, *** *p* < 0.001.

Liver injury related to sinusoidal congestion and hepatocellular vacuolization and necrosis was assessed according to the Suzuki score. While significant differences were not observed among the three groups after 24 h (75H 6 (4–7), 90H 7 (6–8), 90H + SST 5 (4–5), *p* = 0.06), histological injury was higher in 90H (7 (6–9)) relative to 75H (3 (2–5), *p* < 0.001) and 90H + SST (4 (3–5), *p* = 0.002) at 5 days (Figure 2).

### 3.3. Hepatic Regeneration

**Figure 3:**
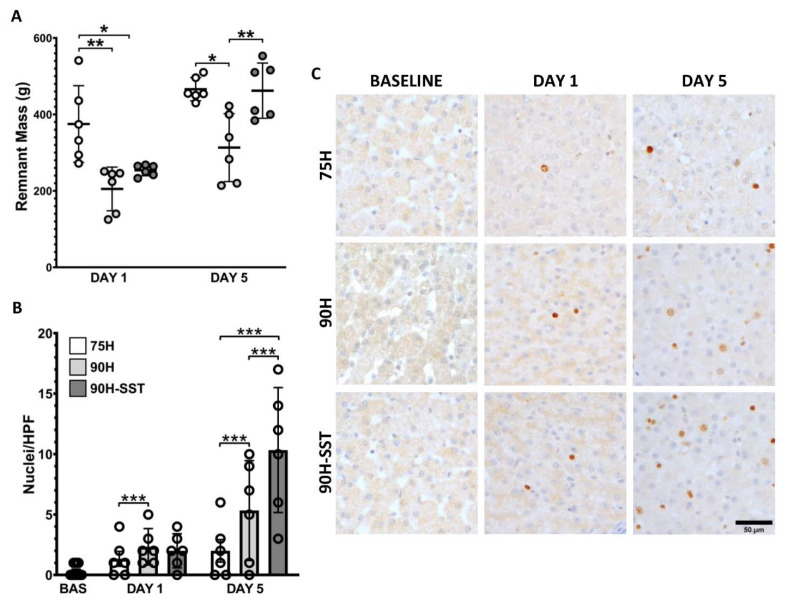
Remnant liver mass measured 1 and 5 days after 75% hepatectomy (75H), 90% hepatectomy (90H), and 90% hepatectomy with continuous perioperative somatostatin infusion (90H + SST) (**A**). Proliferating hepatocellular nuclei evaluated with Ki-67 immunostaining (**B**,**C**). * *p* < 0.05, ** *p* < 0.01, *** *p* < 0.001.

Twenty-four hours after surgery, remnant liver mass was higher in 75H (375 ± 112 g) compared with 90H and 90H + SST (205 ± 57 g and 254 ± 16 g, respectively, *p* < 0.01). In contrast, 5 days after surgery, remnant liver mass was higher in 75H and 90H + SST relative to 90H (469 ± 33 g and 463 ± 79 g versus 313 ± 107, respectively, *p* = 0.003) (Figure 3A).

Proliferating hepatocytes were evaluated with Ki-67 immunostaining. Twenty-four hours after liver resection, 90H and 90H + SST had greater hepatocellular proliferative activity relative to 75H (2 ± 2 HPF^−1^ and 2 ± 2 HPF^−1^ versus 1 ± 1 HPF^−1^, respectively, *p* < 0.001). On the fifth day, Ki-67 immunostaining was higher in all groups relative to 24 h and higher in 90H + SST relative to both 75H and 90H (10 ± 7 HPF^−1^ versus 2 ± 2 HPF^−1^ and 5 ± 6 HPF^−1^, respectively, *p* < 0.001) (Figure 3B,C).

### 3.4. Hepatocellular Apoptosis

**Figure 4:**
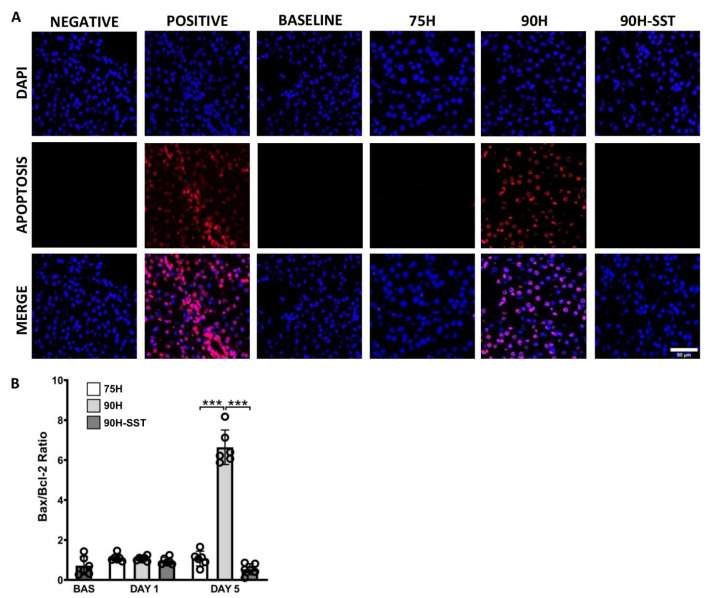
Immunofluorescence of DAPI-stained nuclei (blue) and apoptotic TUNEL-positive nuclei (red) 5 days after 75% hepatectomy (75H), 90% hepatectomy (90H), and 90% hepatectomy with continuous perioperative somatostatin infusion (90H + SST) (**A**). Expression of pro- and anti-apoptotic proteins Bax and Bcl-2 (**B**). *** *p* < 0.001.

TUNEL immunofluorescence was used to evaluate apoptotic cells. After 5 days, a significantly greater number of TUNEL-staining cells were observed in the 90H group relative to 75H and 90H + SST (Figure 4A).

Bax and Bcl-2 are pro- and anti-apoptotic proteins, respectively. The Bax/Bcl-2 protein ratio was analyzed to evaluate the extent of programmed cell death. While there were no significant differences among the three groups at 24 h (*p* = 0.23), Bax/Bcl-2 was significantly higher at 5 days in the 90H group compared with the other two (*p* < 0.001) (Figure 4B).

### 3.5. Extracellular Matrix Remodeling, Hepatic Stellate Cell Activation, and Collagen Deposition

**Figure 5:**
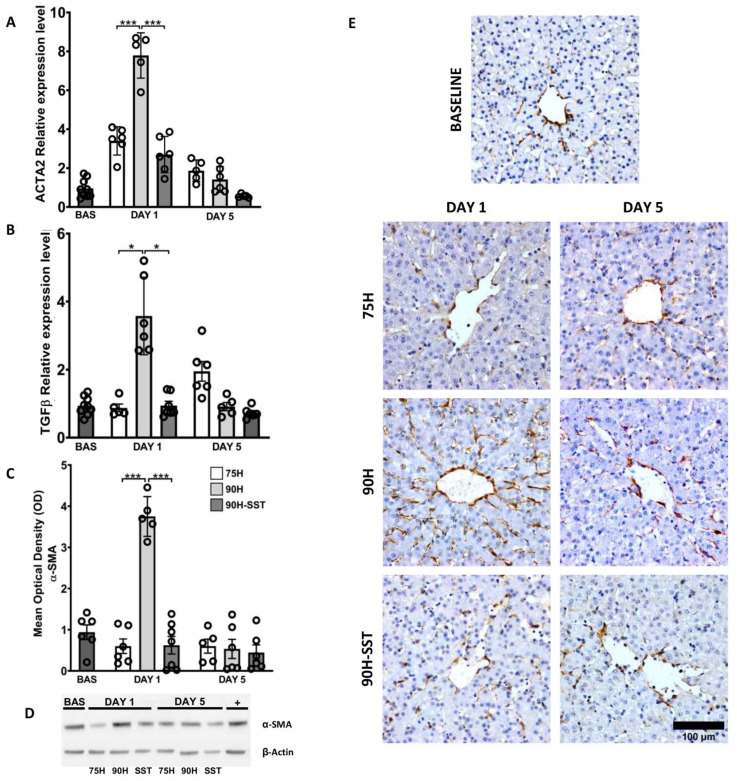
Changes related to hepatic stellate cell activation and transdifferentiation, determined by mRNA expression of ACTA2 (**A**) and TGF-β1 (**B**) and protein expression of α-SMA on Western blot (**C**,**D**) and immunostaining (**E**) following 75% hepatectomy (75H), 90% hepatectomy (90H), and 90% hepatectomy with continuous perioperative somatostatin infusion (90H + SST). * *p* < 0.05, *** *p* < 0.001.

Expression of ACTA2 and TGF-β1 mRNA was higher at 24 h relative to baseline, and it was higher in 90H relative to 75H and 90H + SST for both genes (ACTA2 *p* < 0.001, TGF-β1 *p* = 0.03) (Figure 5A,B). On Western blot analysis, expression of α-SMA protein at 24 h was significantly higher in 90H compared with baseline and 75H and 90H + SST at the same time point (*p* < 0.001 in all cases) (Figure 5C,D). By the fifth postoperative day, all the aforementioned values had returned to baseline levels. Likewise, 24 h after surgery, immunostaining for α-SMA was significantly higher among all groups compared with baseline (*p* < 0.001) and higher in 90H (10.1 ± 2.5) compared with the other two groups (75H 2.8 ± 2.9, 90H + SST 3.3 ± 1.7, *p* < 0.001 for both comparisons) (Figure 5E). On the fifth postoperative day, immunostaining was lowest in 90H + SST (1.2 ± 0.03) and significantly lower at that point compared with the other two groups (75H 3.6 ± 1.6, *p* = 0.005; 90H 3.1 ± 0.8, *p* = 0.001).

**Figure 6:**
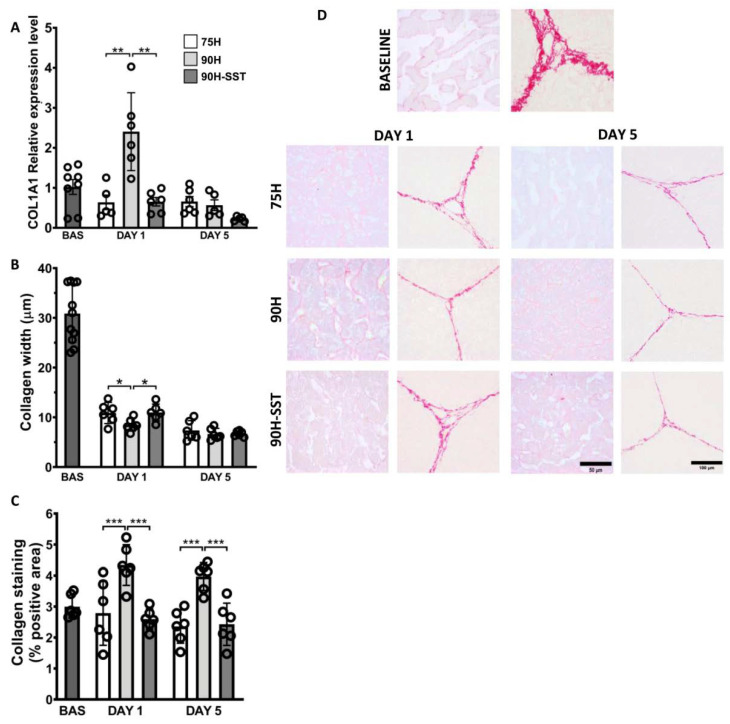
Collagen deposition and extracellular matrix remodeling, determined by mRNA expression of collagen chain type 1 alpha 1 (**A**), collagen width (**B**), and Picro Sirius Red staining of perisinusoidal collagen deposition (**C**,**D**) following 75% hepatectomy (75H), 90% hepatectomy (90H), and 90% hepatectomy with continuous perioperative somatostatin infusion (90H + SST). * *p* < 0.05, ** *p* < 0.01, *** *p* < 0.001.

mRNA expression of COL1A1 at 24 h was significantly higher in the 90H group relative to baseline (*p* = 0.03) and in the former relative to the 75H and 90H + SST groups at the same time point (*p* = 0.005 and *p* = 0.003, respectively) (Figure 6A).

Extracellular matrix remodeling was reflected in changes in the width of the ECM within hepatic lobules. After 24 h and 5 days, ECM width was considerably reduced relative to baseline in all three groups (*p* < 0.001). After 24 h, ECM width was significantly less in 90H (8.1 ± 1.3 μm) relative to 75H (10.7 ± 2.1 μm, *p* = 0.04) and 90H + SST (10.7 ± 1.7 μm, *p* = 0.01), though no differences were detected among the groups on the fifth day (Figure 6C).

After 24 h, tissue samples from the 90H group were notable for significantly greater PSR staining of collagen in the perisinusoidal spaces relative both to baseline (*p* < 0.001) and the 75H and 90H + SST groups at the same time point (*p* < 0.001 for both comparisons). This difference persisted at 5 days (*p* = 0.04) (Figure 6B,D).

### 3.6. Liver Sinusoidal Endothelial Cell Injury

**Figure 7:**
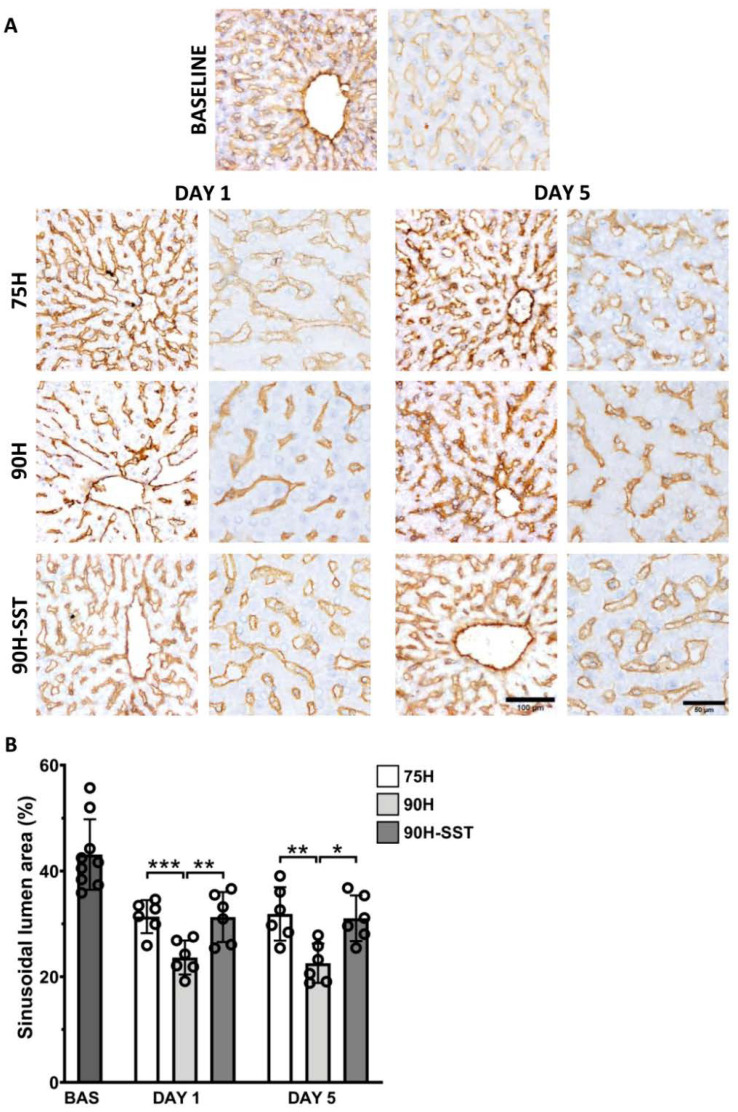
Changes in the CD-31 immunostained liver sinusoidal endothelial cells (**A**) and sinusoidal lumen area (**B**) following 75% hepatectomy (75H), 90% hepatectomy (90H), and 90% hepatectomy with continuous perioperative somatostatin infusion (90H + SST). * *p* < 0.05, ** *p* < 0.01, *** *p* < 0.001.

Liver sinusoidal endothelial cell injury and contraction was analyzed by calculating the sinusoidal lumen area (SLA) in CD31-immunostained samples. Twenty-four hours after surgery, sinusoidal contraction was observed in all three groups relative to baseline (*p* < 0.001). At 24 h, 90H had the lowest SLA (23.5 ± 3.6%) relative to 75H (31.1 ± 3.5%, *p* < 0.001) and 90H + SST (30.9 ± 4.3%, *p* = 0.008). This trend continued on the fifth postoperative day (90H 23.5 ± 3.6% versus 75H 30.9 ± 5.0%, *p* = 0.007, and 30.5 ± 4.3%, *p* = 0.02) (Figure 7).

## 4. Discussion

Herein, we evaluated hemodynamic, biochemical, histological, and molecular changes associated with major (75%) and extended major (90%) hepatectomy in pigs and effects of perioperative somatostatin treatment in this setting. Immediately following the completion of 90% hepatectomy, PVPG was significantly increased in untreated animals. This was followed by increased hepatocellular injury and LSEC injury and contraction 24 h later, both changes that persisted at 5 days. An initial increase in proliferating hepatocytes was observed at 24 h in 90H relative to 75H, though a change in the opposite direction was observed after 5 days, associated with greater apoptosis in the former (90H) group. In relation to increased HSC activation at 24 h, collagen chain type 1 alpha 1 gene expression was upregulated at 24 h in 90H, and increased perisinusoidal collagen deposition was observed both at 24 h and 5 days. In contrast, when somatostatin was administered continuously in the perioperative period, PVPG remained stable at the end of surgery, and less injury in hepatocytes and LSECs was observed at both 24 h and 5 days. Significantly more proliferating hepatocytes were observed at 5 days, and relevant apoptosis was not observed. As well, LSEC contraction and HSC activation were both significantly decreased, and perisinusoidal collagen deposition was not detected.

While the effects of pre-existing fibrosis/cirrhosis on post-hepatectomy outcomes have been studied [22,23,24], little-to-no literature exists regarding the role that major hepatectomy itself plays in initiating the early pathways of fibrogenesis and chronic liver injury. As far as we have been able to encounter in the literature, this is one of few studies (potentially the first) describing early changes of fibrogenesis (e.g., collagen deposition) as early as 24 h after extended major hepatectomy.

Various inciting factors (alcohol abuse, hepatitis C virus infection, cholestasis, metabolic syndrome, etc.) have been described to lead to a common pathway of injury in the liver. Inflammatory infiltrates and apoptosis of damaged hepatocytes stimulate fibrogenic mediators and transdifferentiation of quiescent HSCs into myofibroblast-like cells, provoking contraction and loss of fenestrations in LSECs, degradation of the ECM, fibrogenesis, and angiogenesis [25,26,27]. Until this point, liver resection has largely been excluded from the list of causes of chronic liver injury. There are different reasons for this. As we have seen in this study in the 75H group, major resections leaving a remnant liver of adequate size and quality are not associated with the same changes of injury, sinusoidal collapse, apoptosis, and fibrosis as those seen with an insufficient liver remnant. This is the case for the majority of patients undergoing major hepatectomy in the clinical setting, where different strategies and algorithms have been devised and implemented to avoid insufficient liver remnant and moderate-to-severe (grade B/C) PHLF [7,28,29]. A large proportion of patients that actually do develop relevant PHLF die in the first postoperative weeks to months [30,31], while the greater part of the remainder subsequently succumbs to the oncological processes that were the indication for surgery in the first place [32,33,34,35,36]. For these reasons, very few patients survive for prolonged periods of time with chronic hepatectomy-induced liver failure. If they survive the acute period, some patients may go on to develop hallmarks of cirrhosis and clinically significant portal hypertension (ongoing ascites, development of portosystemic collaterals, gastrointestinal bleeding, etc.).

Somatostatin is actually widely known to inhibit, to a certain degree, “standard” liver regeneration [37,38,39], though its use in the context of extremely SFS livers ultimately has the opposite effect, as we have seen in the present report. Without any form of portal inflow reduction—be it mechanical or pharmacological—the initial regenerative stimulus and, consequentially, response in SFS livers is great (excessive). Given the disproportionate effects of this stimulus on liver cell subpopulations, however, it rapidly becomes detrimental, as it leads to collapse of the sinusoidal lumen by hepatocytes, in the absence of parallel regeneration of non-parenchymal cells (liver sinusoidal endothelial cells, in particular) [17,40,41]. For this reason, somatostatin therapy, which reduces portal vein flow and decreases the initial regenerative stimulus, actually helps maintain regeneration better over the course of several days, due to the fact that it preserves normal hepatic microarchitecture during this process.

Somatostatin and its analogues are known to curb HSC activation [15] and have been seen to attenuate liver fibrosis/fibrogenesis in other forms of liver injury [42,43,44]. Reynaert and colleagues demonstrated that somatostatin administration reduced both gene and protein expression of α-SMA and collagens I and III by HSCs in vitro [45]. It follows that the direct and indirect effects of somatostatin on HSCs can play a beneficial role in mitigating early pathways of HSC activation, collagen production, and early hepatic fibrogenesis following major liver resection, as has been demonstrated in the present report.

**Figure 8:**
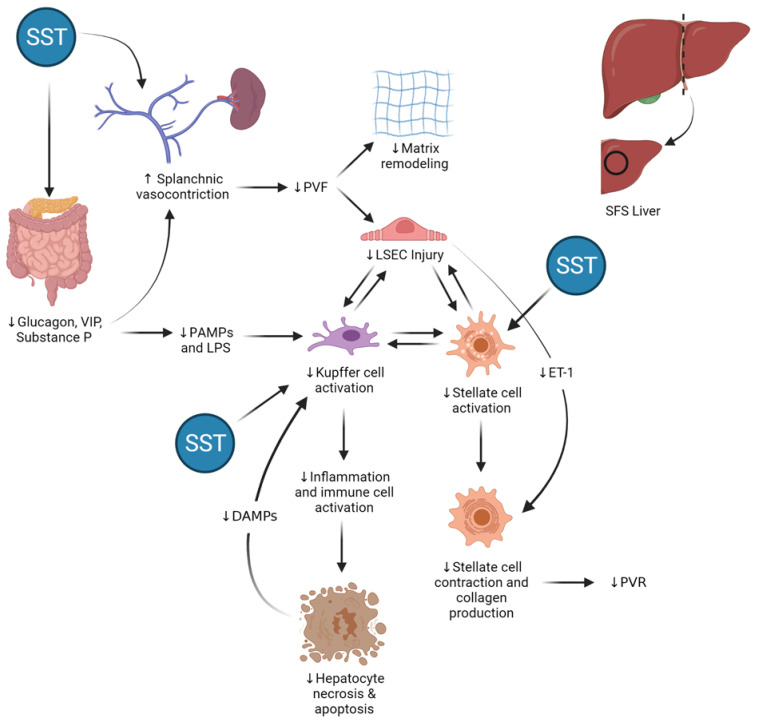
Potential sites of action and effects of somatostatin (SST) therapy in the “small-for-size” (SFS) liver. By binding its receptors in the endothelium and inhibiting secretion of gut-derived vasodilatory peptides, somatostatin (SST) induces vasoconstriction in mesenteric arteries and portomesenteric veins and reduces portal vein flow (PVF) in a dose-dependent manner. In the SFS liver, lower PVF results in decreased mechanical shear stress in the extracellular matrix and liver sinusoidal endothelial cells (LSECs) and reduced expression and translation of endothelin-1 (ET-1) mRNA by SECs. Both the indirect effects as well as the direct action of SST on hepatic stellate cells lead to decreased hepatic stellate cell activation and contraction, thereby preventing collagen deposition and helping maintain normal hepatic sinusoidal diameter, reducing portal vein resistance (PVR) and improving hepatic microcirculatory flow. At the same time, SST reduces intestinal permeability, leading to lower circulating levels of gut-derived pathogen-associated molecular patterns (PAMPs) and lipopolysaccharides (LPS). These multiple actions lead to decreased activation of Kupffer cells in the hepatic sinusoids, thereby stemming the production and secretion of different acute-phase molecules, such tumor necrosis factor alpha (TNF-α) and Fas ligand, which are inducers of hepatocellular necrosis as well as programmed cell death.

Figure 8 provides an overview of the potential sites and mechanisms of action of somatostatin in a “small-for-size” liver following major reduction in mass. The actions of somatostatin on the gut and portomesenteric vasculature serve to lower portal inflow, resulting in less sinusoidal shear stress and decreased activation of injury pathways that may ultimately lead to parenchymal necrosis and apoptosis. At the same time, somatostatin acts to reduce hepatic stellate cell activation directly, reducing contraction and abrogating aforementioned early pathways of fibrogenesis in the regenerating liver.

The present study has limitations concerned with its experimental nature and the fact that it evaluates only healthy livers and not ones suffering from chronic disease processes, such as NASH or cirrhosis. Nonetheless, the effects of somatostatin have been studied in different animal models, and it has been seen that treatment with somatostatin and its analogues reduces inflammation and fibrosis among liver with chronic injury, as well [45]. While clinical corroboration of the results of this study is still needed, some evidence has been published to date describing the clinical use of SST for portal inflow modulation and PHLF/SFSS prevention. In a single center randomized controlled trial, SST was administered to patients with clinically significant portal hypertension undergoing liver transplantation with whole grafts. Patients were randomized 2:1 to perioperative SST administered during five days (N = 18) versus placebo (N = 11). The primary endpoint—>20% reduction in HVPG in response to SST bolus—was met in 55% of SST-treated patients and none receiving the placebo. Though the trial was not powered to do so, no differences in major clinical outcome measures (adverse events, long-term complications, or graft and patient survival rates) were detected [46]. In another clinical pilot study, SST was initiated intraoperatively following completion of major or extended major hepatectomy when post-resection portal pressure was >20 mmHg. The somatostatin bolus (250 µg) immediately reduced portal pressure in the majority of cases and was maintained as a continuous 250-µg/h infusion given over the course of five days [47]. In a recent update on this series, portal inflow modulation using SST was administered in 31 patients undergoing major or extended major hepatectomy for oncological indications. Among them, five (16%) met PHLF criteria, though neither grades nor outcomes were reported [48].

## 5. Conclusions

In this preclinical model of extended major hepatectomy, perioperative somatostatin administration modified portal pressure, injury, apoptosis, and HSC activation, stemming early changes related to hepatic fibrogenesis that were seen in liver remnants not receiving SST. While more data from longer-term animal and human studies are needed, the results of this study indicate a role for this treatment in the avoidance of PHLF and its complications as well as longer-term consequences of hepatectomy-induced liver injury.

## Figures and Tables

**Table 1:** Evolution of hepatic hemodynamic parameters measured at baseline, at the end of surgery after major (75H) or extended major (90H, 90H + SST) liver resection, and after surgery at 24 h and 5 days.

Variable	Time Point	75H	90H	90H + SST	*p*
PVF (mL/min)	Baseline	778 (600–950)	833 (600–1100)	910 (650–1010)	0.16
-	End surgery	830 (600–1075)	866 (630–1120)	739 (525–1070)	0.12
-	24 h	938 (750–1100)	881 (700–1050)	868 (700–1050)	0.52
-	5 days	955 (730–1160)	803 (600–900)	830 (600–1000)	0.60
-	Baseline	3 1–4	3 1–5	3 1–5	0.90
PVPG (mmHg)	End surgery	4 2–6	8 3–13	4 2–6	<0.001
-	24 h	3 2–4	4 3–6	4 2–6	0.52
-	5 days	5 4–7	7 6,7	5 2–6	0.20
HAF (mL/min)	Baseline	140 80–190	147 100–190	144 90–175	0.78
-	End surgery	63 30–90	57 25–100	77 30–120	0.08
-	24 h	71 50–90	57 30–90	65 30–90	0.57
-	5 days	78 50–115	57 38–75	82 35–140	0.45

## Data Availability

The data that support the findings of this study are available on reasonable request from the corresponding author.

## References

[1] Sung H., Ferlay J., Siegel R.L., Laversanne M., Soerjomataram I., Jemal A., Bray F. (2021). Global cancer statistics 2020: GLOBOCAN estimates of incidence and mortality worldwide for 36 cancers in 185 countries. CA Cancer J. Clin..

[2] Tsilimigras D.I., Brodt P., Clavien P.-A., Muschel R.J., D’Angelica M.I., Endo I., Parks R.W., Doyle M., de Santibañes E., Pawlik T.M. (2021). Liver metastases. Nat. Rev. Dis. Primers.

[3] Riquelme F., Muñoz C., Ausania F., Hessheimer A.J., Torres F., Calatayud D., Sandomenico R., Pérez R.G., Ferrer J., Fuster J. (2020). Laparoscopic versus open hemihepatectomy: Comprehensive comparison of complications and costs at 90 days using a propensity method. Updates Surg..

[4] Balzan S., Belghiti J., Farges O., Ogata S., Sauvanet A., Delefosse D. (2005). The ”50-50 Criteria” on Postoperative Day 5. Ann. Surg..

[5] Dahm F., Georgiev P., Clavien P.-A. (2005). Small-for-Size Syndrome After Partial Liver Transplantation: Definition, Mechanisms of Disease and Clinical Implications. Am. J. Transplant..

[6] Broek M.A.J.V.D., Damink S.W.M.O., Dejong C.H.C., Lang H., Malagó M., Jalan R., Saner F.H. (2008). Liver failure after partial hepatic resection: Definition, pathophysiology, risk factors and treatment. Liver Int..

[7] Rahbari N.N., Garden O.J., Padbury R., Brooke-Smith M., Crawford M., Adam R., Koch M., Makuuchi M., Dematteo R.P., Christophi C. (2011). Posthepatectomy liver failure: A definition and grading by the International Study Group of Liver Surgery (ISGLS). Surgery.

[8] Eguchi S., Yanaga K., Sugiyama N., Okudaira S., Furui J., Kanematsu T. (2003). Relationship between portal venous flow and liver regeneration in patients after living donor right-lobe liver transplantation. Liver Transplant..

[9] Maetani Y., Itoh K., Egawa H., Shibata T., Ametani F., Kubo T., Kiuchi T., Tanaka K., Konishi J. (2003). Factors influencing liver regeneration following living-donor liver transplantation of the right hepatic lobe. Transplantation.

[10] Hessheimer A.J., Fondevila C., Taurá P., Muñoz J., Sánchez O., Fuster J., Rimola A., García-Valdecasas J.C. (2011). Decompression of the Portal Bed and Twice-Baseline Portal Inflow Are Necessary for the Functional Recovery of a “Small-for-Size” Graft. Ann. Surg..

[11] Ku Y., Fukumoto T., Nishida T., Tominaga M., Maeda I., Kitagawa T., Takao S., Shiotani M., Tseng A., Kuroda Y. (1995). Evidence that portal vein decompression improves survival of canine quarter orthotopic liver transplantation. Transplantation.

[12] Emond J.C., Renz J.F., Ferrell L.D., Rosenthal P., Lim R.C., Roberts J.P., Lake J., Ascher N.L. (1996). Functional Analysis of Grafts from Living Donors. Ann. Surg..

[13] Troisi R., Ricciardi S., Smeets P., Petrovic M., Van Maele G., Colle I., Van Vlierberghe H., De Hemptinne B. (2005). Effects of Hemi-Portocaval Shunts For Inflow Modulation on the Outcome of Small-for-Size Grafts in Living Donor Liver Transplantation. Am. J. Transplant..

[14] Fondevila C., Hessheimer A.J., Taurá P., Sanchez O., Calatayud D., De Riva N., Munoz J., Fuster J., Rimola A., García-Valdecasas J.C. (2010). Portal hyperperfusion: Mechanism of injury and stimulus for regeneration in porcine small-for-size transplantation. Liver Transplant..

[15] Hessheimer A.J., Escobar B., Muñoz J., Flores-Villalba E., Gracia-Sancho J., Taurá P., Fuster J., Rimola A., García-Valdecasas J.C., Fondevila C. (2014). Somatostatin Therapy Protects Porcine Livers in Small-for-Size Liver Transplantation. Am. J. Transplant..

[16] Ninomiya M., Shirabe K., Terashi T., Ijichi H., Yonemura Y., Harada N., Soejima Y., Taketomi A., Shimada M., Maehara Y. (2010). Deceleration of Regenerative Response Improves the Outcome of Rat with Massive Hepatectomy. Am. J. Transplant..

[17] Hessheimer A.J., Martínez de la Maza L., Adel Al Shwely F., Espinoza A.S., Ausania F., Fondevila C. (2019). Somatostatin and the “small-for-size”. Liver Int. J. Mol. Sci..

[18] Court F.G., Wemyss-Holden S.A., Morrison C.P., Teague B.D., Laws P.E., Kew J., Dennison A.R., Maddern G.J. (2003). Segmental nature of the porcine liver and its potential as a model for experimental partial hepatectomy. Br. J. Surg..

[19] Court F.G., E Laws P., Morrison C.P., Teague B.D., Metcalfe M.S., A Wemyss-Holden S., Dennison A.R., Maddern G.J. (2004). Subtotal hepatectomy: A porcine model for the study of liver regeneration. J. Surg. Res..

[20] Mohkam K., Darnis B., Mabrut J.-Y. (2016). Porcine models for the study of small-for-size syndrome and portal inflow modulation: Literature review and proposal for a standardized nomenclature. J. Hepato Biliary Pancreat. Sci..

[21] Suzuki S., Nakamura S., Koizumi T., Sakaguchi S., Baba S., Muro H., Fujise Y. (1991). The beneficial effect of a prostaglandin 12 analog on ischemic rat liver. Transplantation.

[22] Aierken Y., Kong L.-X., Li B., Liu X.-J., Lu S., Yang J.-Y. (2020). Liver fibrosis is a major risk factor for liver regeneration. Medicine.

[23] Akiyama T., Miyamoto Y., Imai K., Yamashita Y., Nomoto D., Daitoku N., Sakamoto Y., Kiyozumi Y., Tokunaga R., Eto K. (2020). Fibrosis-4 Index, a Noninvasive Fibrosis Marker, Predicts Survival Outcomes after Hepatectomy for Colorectal Cancer Liver Metastases. Ann. Surg. Oncol..

[24] Serenari M., Han K.-H., Ravaioli F., Kim S.-U., Cucchetti A., Han D.-H., Odaldi F., Ravaioli M., Festi D., Pinna A.D. (2020). A nomogram based on liver stiffness predicts postoperative complications in patients with hepatocellular carcinoma. J. Hepatol..

[25] Bataller R., Brenner D.A. (2005). Science in medicine Liver fibrosis. J. Clin. Investig..

[26] Puche J.E., Lee Y.A., Jiao J., Aloman C., Fiel M.I., Muñoz U., Kraus T., Lee T., Yee H.F., Friedman S.L. (2013). A novel murine model to deplete hepatic stellate cells uncovers their role in amplifying liver damage in mice. Hepatology.

[27] Gracia-Sancho J., Maeso-Díaz R., Fernández-Iglesias A., Navarro-Zornoza M., Bosch J. (2015). New cellular and molecular targets for the treatment of portal hypertension. Hepatol. Int..

[28] Kishi Y., Vauthey J.-N. (2021). Issues to be considered to address the future liver remnant prior to major hepatectomy. Surg. Today.

[29] Søreide J.A., Deshpande R. (2021). Post hepatectomy liver failure (PHLF) – Recent advances in prevention and clinical management. Eur. J. Surg. Oncol..

[30] Jara M., Reese T., Malinowski M., Valle E., Seehofer D., Puhl G., Neuhaus P., Pratschke J., Stockmann M. (2015). Reductions in post-hepatectomy liver failure and related mortality after implementation of the LiMAx algorithm in preoperative work-up: A single-centre analysis of 1170 hepatectomies of one or more segments. HPB.

[31] Gilg S., Sandström P., Rizell M., Lindell G., Ardnor B., Stromberg C., Isaksson B. (2018). The impact of post-hepatectomy liver failure on mortality: A population-based study. Scand. J. Gastroenterol..

[32] Nuzzo G., Giuliante F., Ardito F., Giovannini I., Aldrighetti L., Belli G., Bresadola F., Calise F., Valle R.D., D’Amico D.F. (2012). Improvement in Perioperative and Long-term Outcome After Surgical Treatment of Hilar Cholangiocarcinoma. Arch. Surg..

[33] Nagino M., Ebata T., Yokoyama Y., Igami T., Sugawara G., Takahashi Y. (2013). Evolution of surgical treatment for perihilar cholangiocarcinoma: A single-center 34-year review of 574 consecutive resections. Ann. Surg..

[34] Franken L.C., Schreuder A.M., Roos E., van Dieren S., Busch O.R., Besselink M.G., van Gulik T.M. (2019). Morbidity and mortality after major liver resection in patients with perihilar cholangiocarcinoma: A systematic review and meta-analysis. Surgery.

[35] Oldhafer K.J., Donati M., Jenner R.M., Stang A., Stavrou G.A. (2014). ALPPS for Patients with Colorectal Liver Metastases: Effective Liver Hypertrophy, but Early Tumor Recurrence. World J. Surg..

[36] Adam R., Imai K., Castro-Benitez C., Allard M., Vibert E., Cunha A.S., Cherqui D., Baba H., Castaing D. (2016). Outcome after associating liver partition and portal vein ligation for staged hepatectomy and conventional two-stage hepatectomy for colorectal liver metastases. BJS.

[37] Kokudo N., Kothary P.C., Eckhauser F.E., Raper S.E. (1991). Inhibitory effects of somatostatin on rat hepatocyte proliferation are mediated by cyclic AMP. J. Surg. Res..

[38] Kokudo N., Kothary P.C., Eckhauser F.E., Nakamura T., Raper S.E. (1992). Inhibition of DNA synthesis by somatostatin in rat hepatocytes stimulated by hepatocyte growth factor or epidermal growth factor. Am. J. Surg..

[39] Kothary P.C., Kokudo N., Eckhauser F.E., Delvalle J., Raper S.E. (1995). Preferential suppression of insulin-stimulated proliferation of cultured hepatocytes by somatostatin: Evidence for receptor-mediated growth regulation. J. Cell. Biochem..

[40] Michalopoulos G.K., DeFrances M.C., Cressman D.E., Greenbaum L.E., DeAngelis R.A., Ciliberto G., Furth E.E., Poli V., Taub R. (1997). Liver Regeneration. Science.

[41] Taub R. (2004). Liver regeneration: From myth to mechanism. Nat. Rev. Mol. Cell Biol..

[42] Aziz N.M., Ragy M.M., Ahmed S.M. (2018). Somatostatin analogue, Octreotide, improves restraint stress-induced liver injury by ameliorating oxidative stress, inflammatory response, and activation of hepatic stellate cells. Cell Stress Chaperon.

[43] Zhang C., Liu X.-Q., Sun H.-N., Meng X.-M., Bao Y.-W., Zhang H.-P., Pan F.-M., Zhang C. (2018). Octreotide attenuates hepatic fibrosis and hepatic stellate cells proliferation and activation by inhibiting Wnt/β-catenin signaling pathway, c-Myc and cyclin D1. Int. Immunopharmacol..

[44] Wang J., Wang L., Song G., Han B. (2013). The mechanism through which octreotide inhibits hepatic stellate cell activity. Mol. Med. Rep..

[45] Reynaert H., Rombouts K., Jia Y., Urbain D., Chatterjee N., Uyama N., Geerts A. (2005). Somatostatin at nanomolar concentration reduces collagen I and III synthesis by, but not proliferation of activated rat hepatic stellate cells. Br. J. Pharmacol..

[46] Troisi R.I., Vanlander A., Giglio M.C., van Limmen J., Scudeller L., Heyse B. (2019). Somatostatin as inflow modulator in liver-transplant recipients with severe portal hypertension: A randomized trial. Ann. Surg..

[47] Rhaiem R., Piardi T., Chetboun M., Pessaux P., Lestra T., Memeo R., Kianmanesh R., Sommacale D. (2018). Portal Inflow Modulation by Somatostatin After Major Liver Resection. Ann. Surg..

[48] Rhaiem R., Zimmermann P., Brustia R., Lhuaire M., Piardi T., Kianmanesh R., Sommacale D. (2021). Somatostatin perfusion and modulation of portal inflow after major liver resection: Response to “Post hepatectomy liver failure (PHLF)-Recent advances in prevention and clinical management”. Eur. J. Surg. Oncol..

